# Supplementary figures and images for: Interpretable machine learning for automated left ventricular scar quantification in hypertrophic cardiomyopathy patients
Source: PLOS Digit Health. 2023 Jan 4;2(1):e0000159. doi: 10.1371/journal.pdig.0000159 (PMC9931226; doi:10.1371/journal.pdig.0000159)

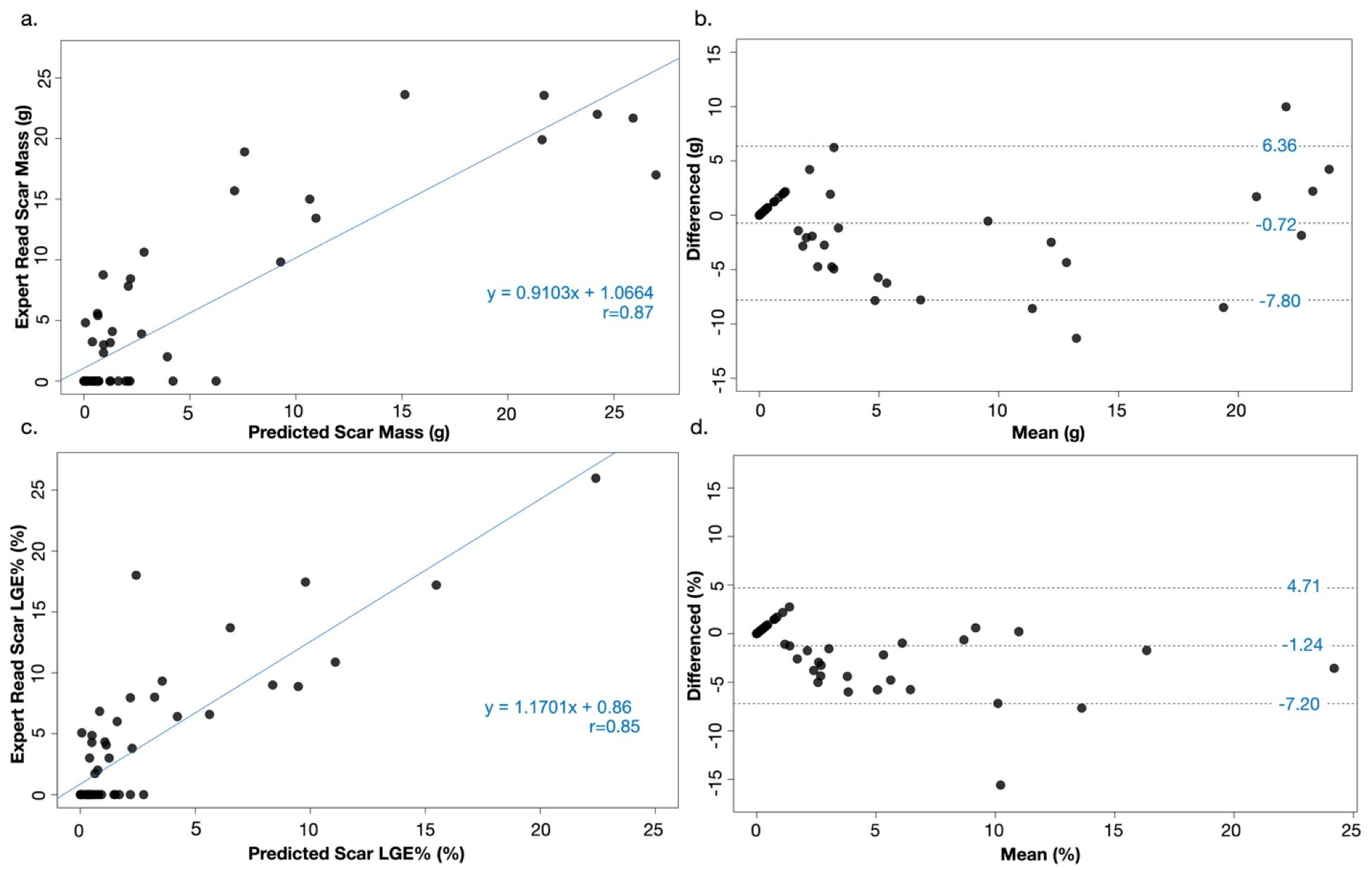

Supplement: S1 Fig — Correlation (a) and Bland-Altman analysis (b) between the expert-based manual analysis and the model prediction for CMR LGE scar mass. Correlation (c) and Bland-Altman analysis (d) between the expert-based manual analysis and model prediction for percentage of LGE volume. (TIFF) [file pdig.0000159.s001.tiff]

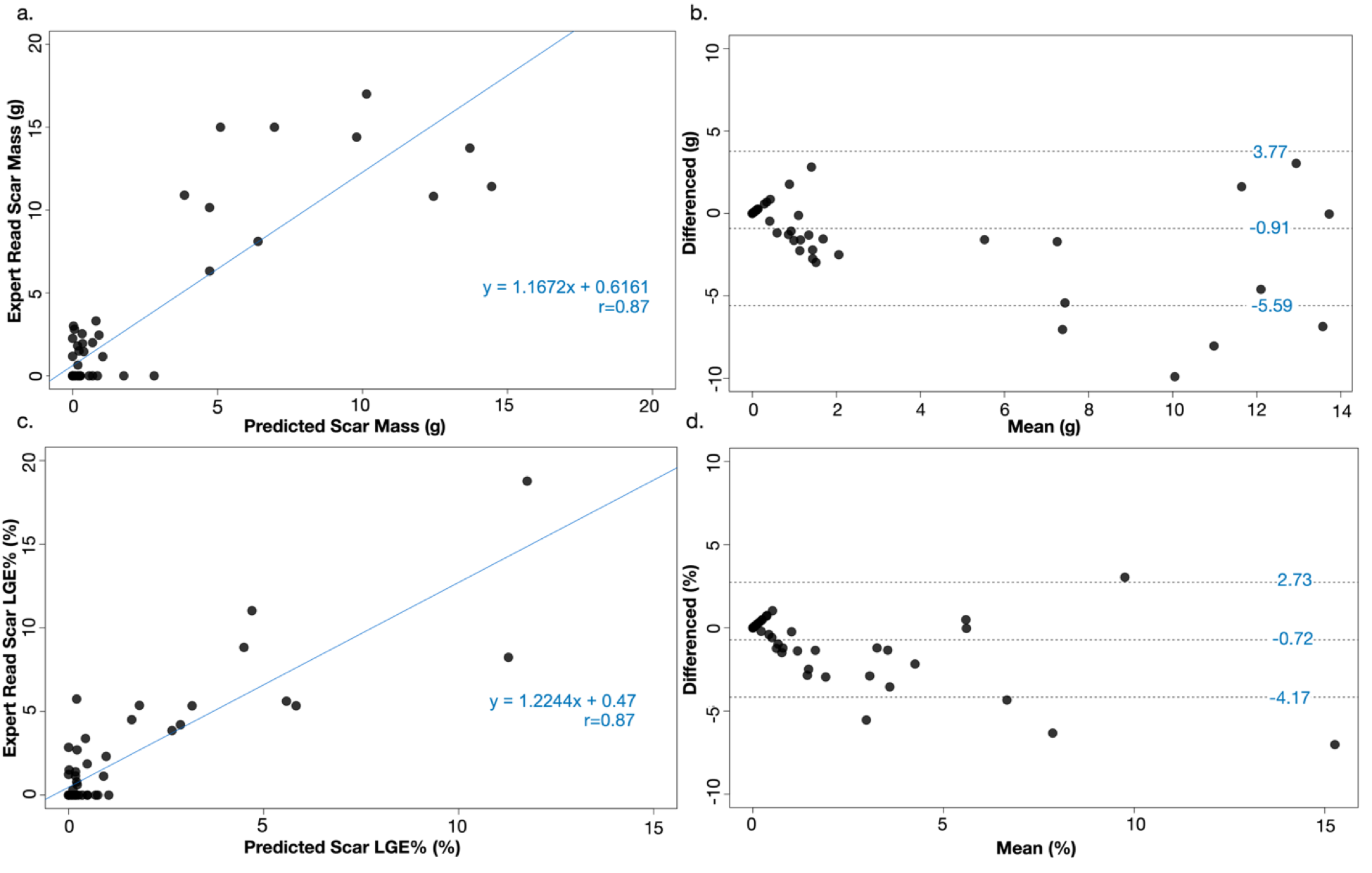

Supplement: S2 Fig — Correlation (a) and Bland-Altman analysis (b) between the expert-based manual analysis and the model prediction for CMR LGE scar mass. Correlation (c) and Bland-Altman analysis (d) between the expert-based manual analysis and model prediction for percentage of LGE volume. (TIFF) [file pdig.0000159.s002.tiff]
