# Supplementary material for: Interpretable machine learning for automated left ventricular scar quantification in hypertrophic cardiomyopathy patients
Source: PLOS Digit Health. 2023 Jan 4;2(1):e0000159. doi: 10.1371/journal.pdig.0000159 (PMC9931226; doi:10.1371/journal.pdig.0000159)
Supplement: Attachment — Submitted filename: ML for LV scar quant in HCM_Plos One review.docx [file pdig.0000159.s006.docx]

**Invited Review**

**Recommendation:** Accept

This is a retrospective study that aimed to train a machine learning model to quantify scar o cardiovascular magnetic resonance late gadolinium enhancement (LGE) images in patients with hypertrophic cardiomyopathy. Numerous studies have shown that scar burden predicts clinical outcomes in these patients; currently, scar quantification in patients with HCM is mostly manually performed by radiologists, making it quite subjective, cumbersome, and error prone. Machine learning models that have been developed for automated scar quantification are limited due to the need for image pre-processing as well as their comparison to a single institutional reader as the reference standard. The authors of this study aimed to address these key limitations in this study.

The authors used approximately 2000 images to train the Shape-attentive U-Net (SAUNet) model, and then tested the model on approximately 500 images. They found that the model showed good correlation for both LV segmentation as well as scar quantification, when compared to manual LV segmentation and scar quantification. Furthermore, the model could efficiently perform these tasks using uncropped images and on a rapid timeframe.

There are several notable strengths to this study. First, the images used to train the model were acquired from multiple sites, vendors, readers, and analysis packages making this study unique and more broadly generalizable. Second, images were not pre-processed, making it more relevant to implementation in clinical practice.

There are a few key limitations. First, the database used to train the model was not large in size. The ~2,000 images used to train the model came from only 247 patients, of which only 200 patients had LGE scar. Other studies (for example, Fahmy 2019 Radiology) included images from over 1,000 patients. Second, only two expert readers were used as the reference standard. This study would be strengthened by expanding the pool of expert readers, particularly given the heterogenous and patchy nature of hypertrophic cardiomyopathy scar.

Overall, quantification of scar on CMR LGE images in patients with HCM is a key prognostication tool in assessing risk of sudden cardiac death. Rapid and accurate scar quantification provides important information to cardiologists managing these patients, and this paper offers a novel machine learning model for scar quantification.
